# Supplementary figures and images for: Differences in the rare variant spectrum among human populations
Source: PLoS Genet. 2017 Feb 1;13(2):e1006581. doi: 10.1371/journal.pgen.1006581 (PMC5310914; doi:10.1371/journal.pgen.1006581)

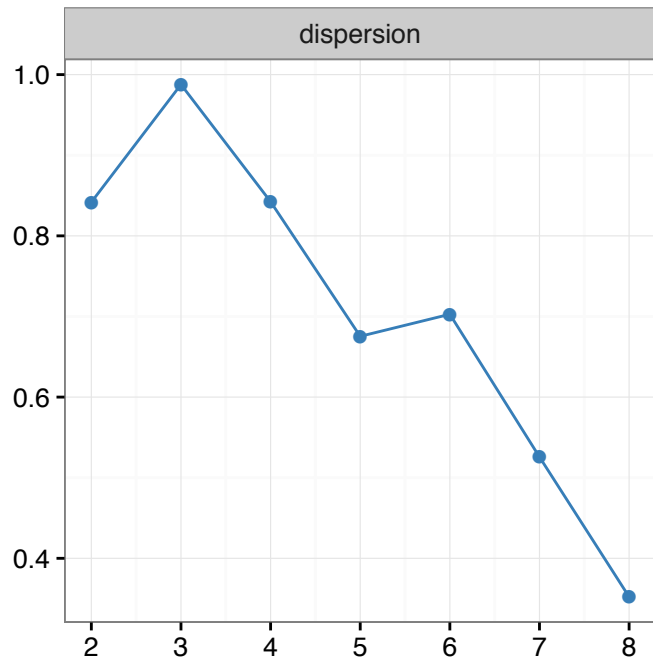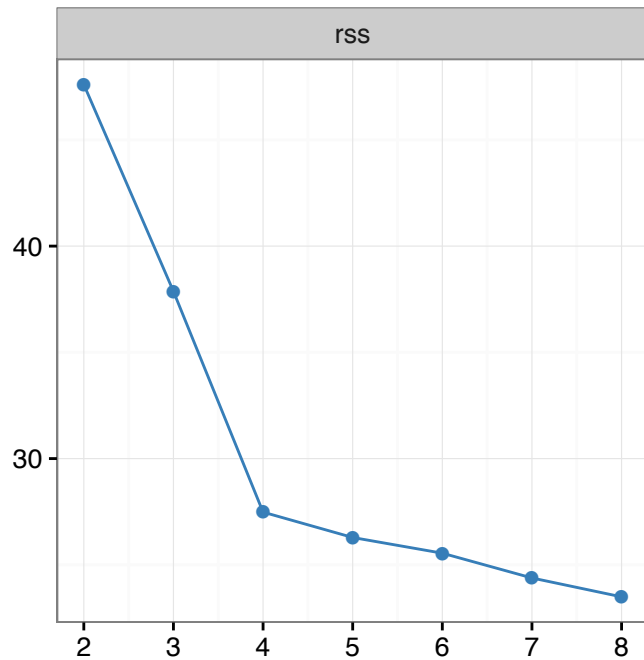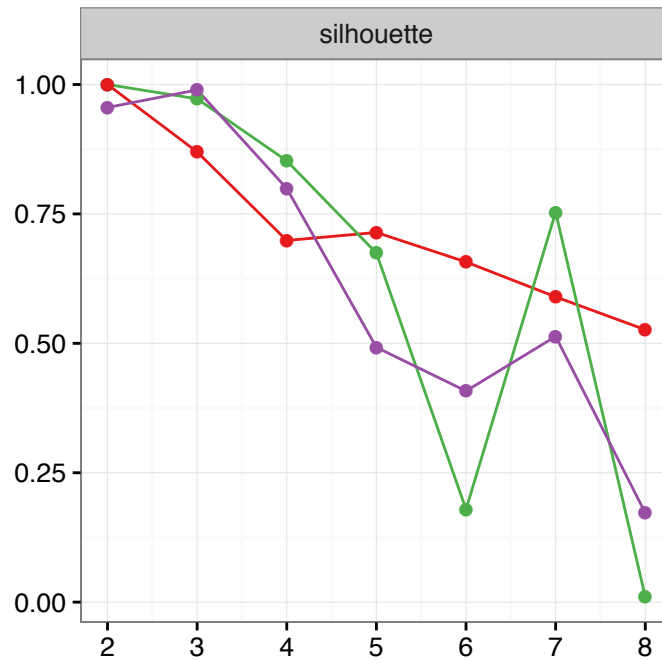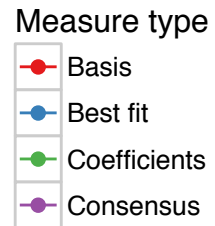

Supplement: S1 Fig — Each plot shows the value of a measure, computed over 50 random start points, for factorization ranks from 2 to 8. From left to right: Dispersion, a measure of reproducibility of clusters across runs (1 = perfectly reproducible); Residual sum of squares (lower = better fit); Silhouette, a measure of how reliably elements can be assigned to clusters (1 = perfectly reliably). (PDF) [file pgen.1006581.s001.pdf]

**A**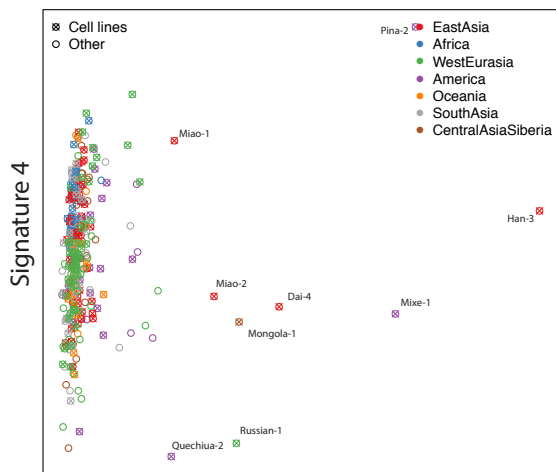**B** Signature 3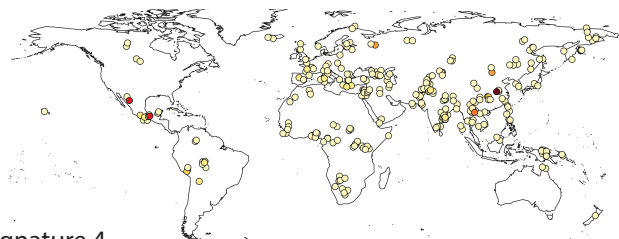

Signature 4

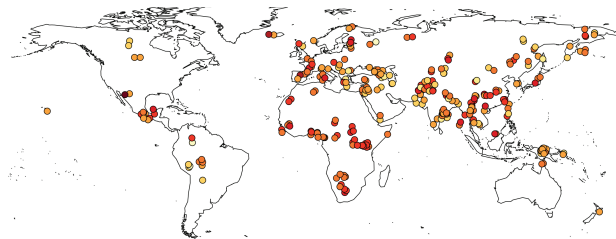**C**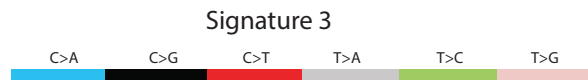**D**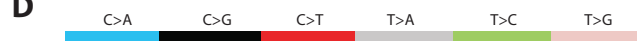

Signature 3

Signature 4

**E**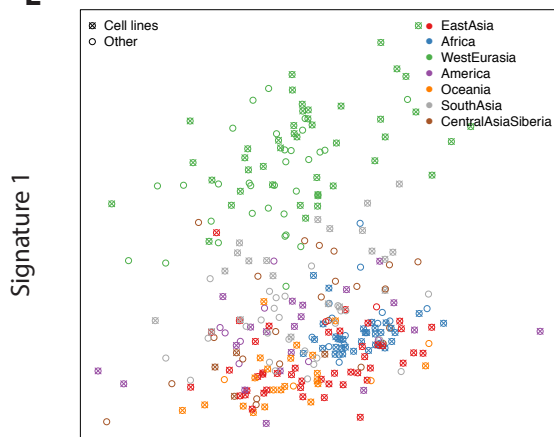

Signature 4

**F**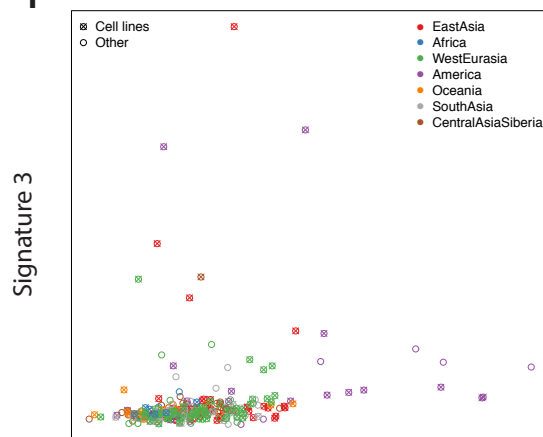

Signature 2

Supplement: S2 Fig — A: Per-sample coefficients for signatures 3 and 4. B: Geographic distribution of signatures 3 and 4. C: Mutational spectrum of signature 3. D: Mutational spectrum of signature 4. E-F: Comparison of loadings of signatures 1 and 2 with signatures 3 and 4. In supplementary plots, we denote the signatures obtained from fr variants with rank k by signaturer,k, so that signature2,4 is equivalent to the signature in the main text. (PDF) [file pgen.1006581.s002.pdf]

**A**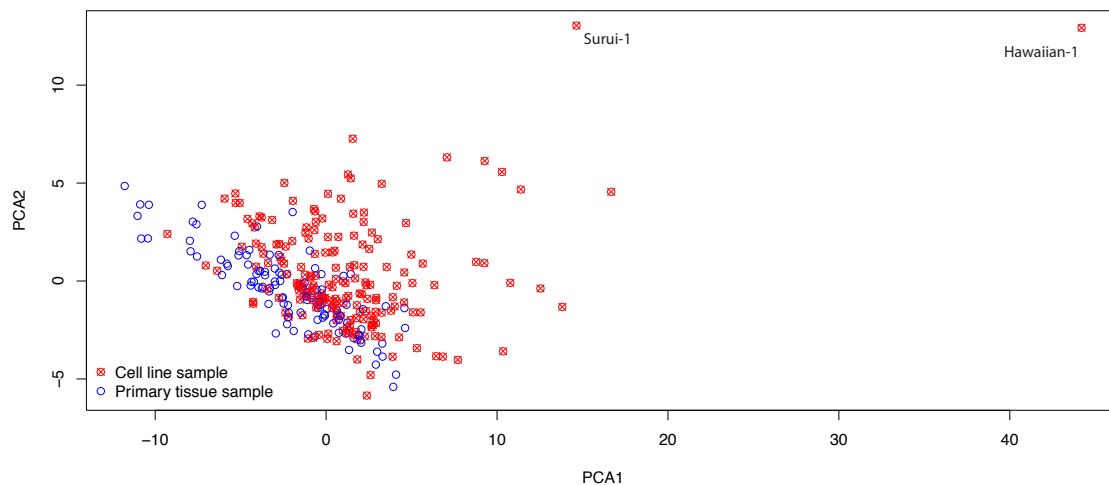**B**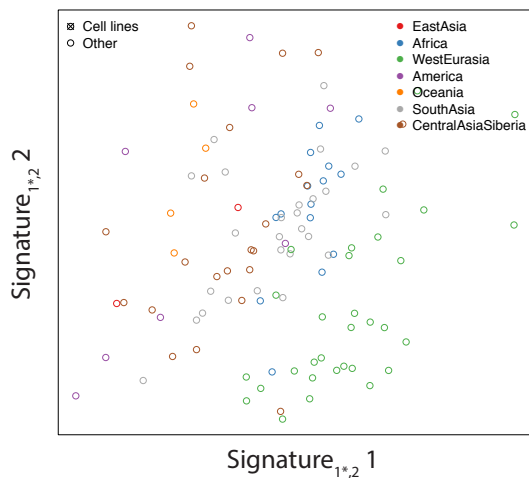**C**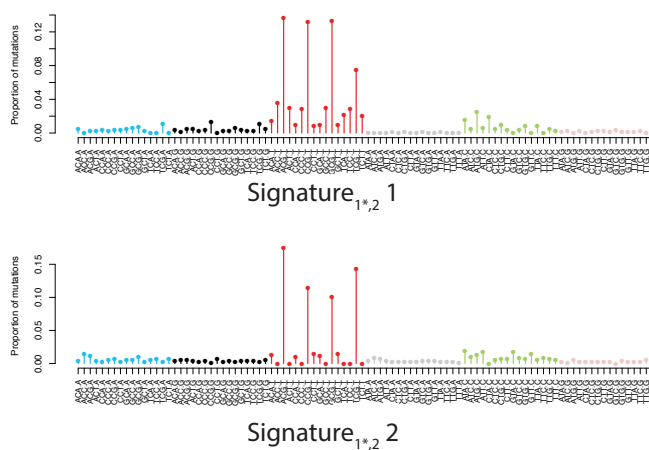

Supplement: S3 Fig — A: The first two principal components of the mutational spectrum of f1 variants, showing the difference between cell line and primary tissue derived samples. B&C: Mutational signatures inferred from f1 variants with rank 2, but excluding cell line samples. B: Factor loadings for signature1*,2 1 and 2 (asterisk denotes no cell lines). C: Mutational signatures1*,2 1 and 2. Signature1*,2 1 is confounded with CpG mutations in this case, but clearly shows an elevated level of TCC>T and ACC>T mutations. (PDF) [file pgen.1006581.s003.pdf]

**A**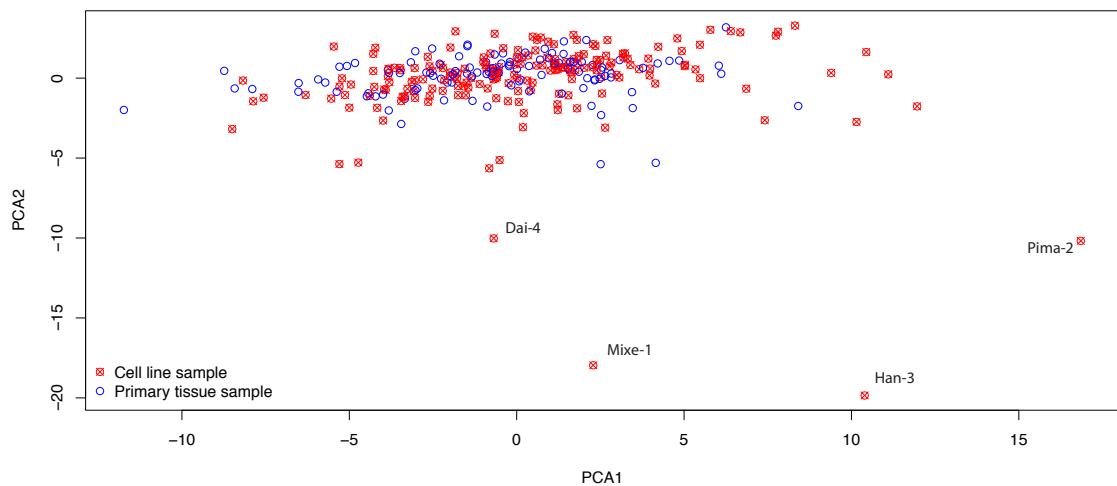**B**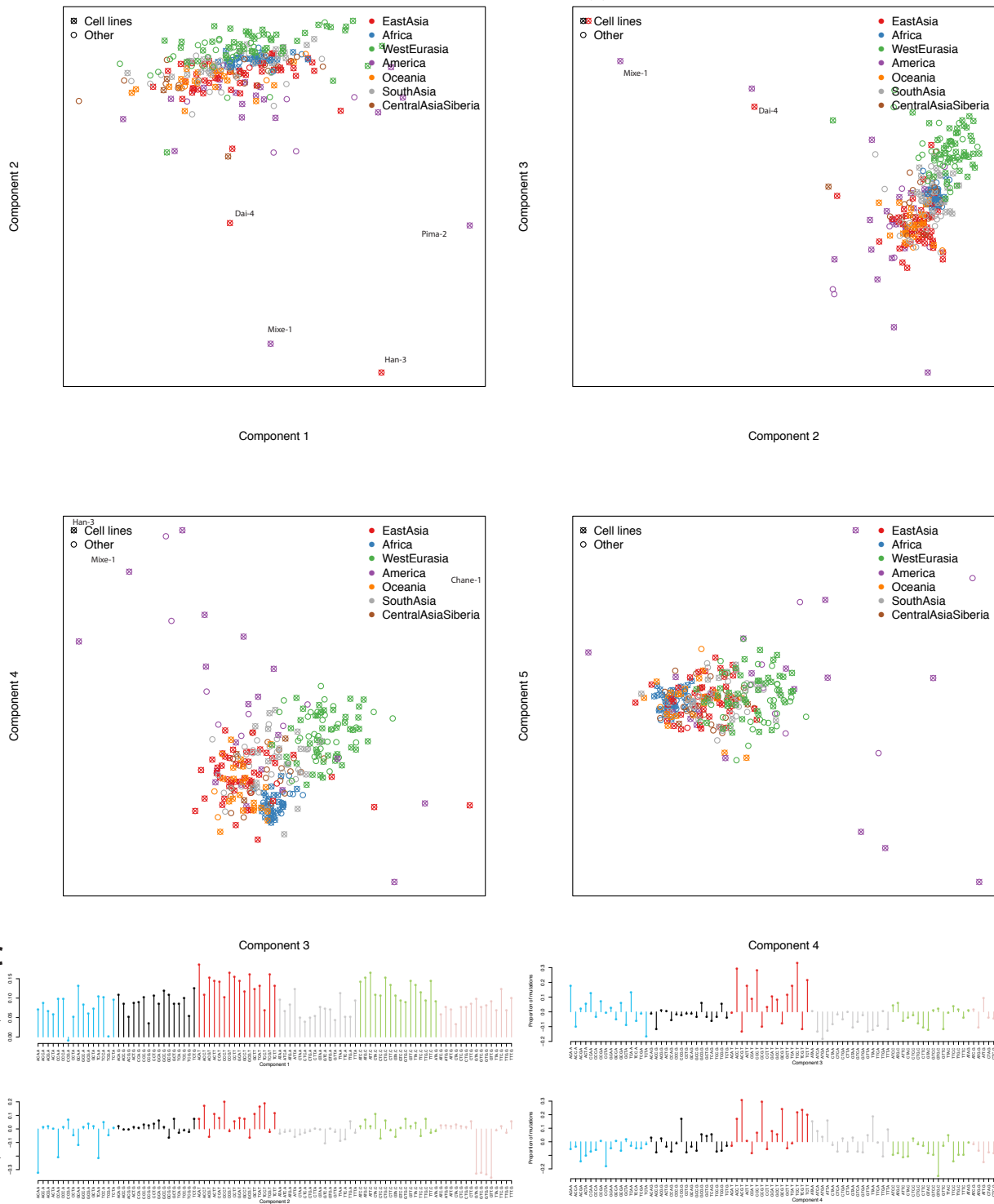**C**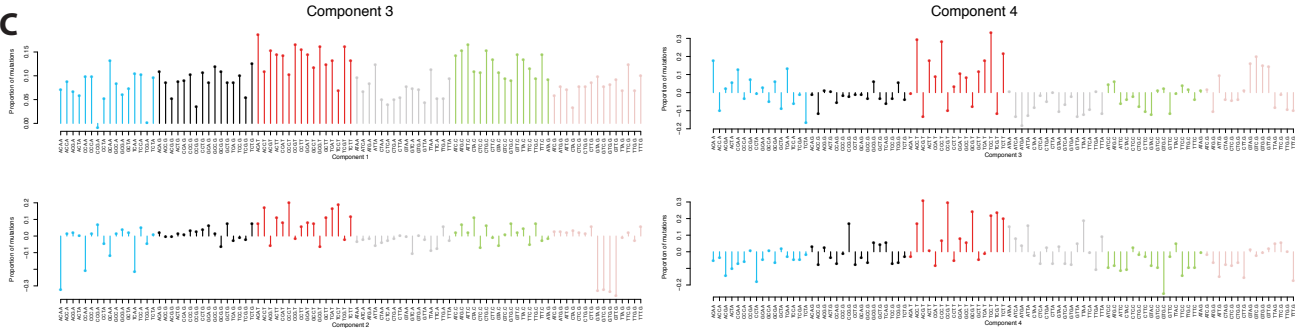

Supplement: S4 Fig — A: The first two principal components of the mutational spectrum of f2 variants, showing no difference between cell line and primary tissue derived samples. B: Principal component positions. Labeled by sample source (A) and geographic region (B). C: Component loadings. Note that principal components 2,3 and 4 correspond roughly to mutational signatures2,4 3, 1 and 2 respectively. (PDF) [file pgen.1006581.s004.pdf]

**A**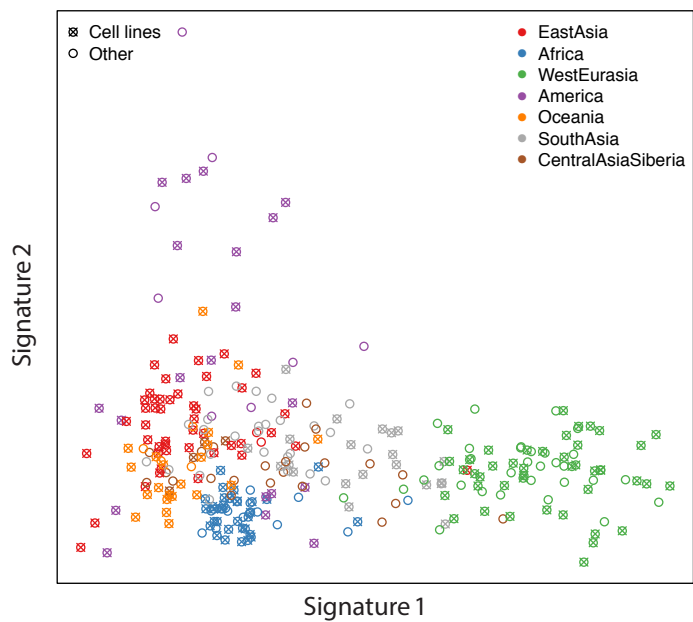**B**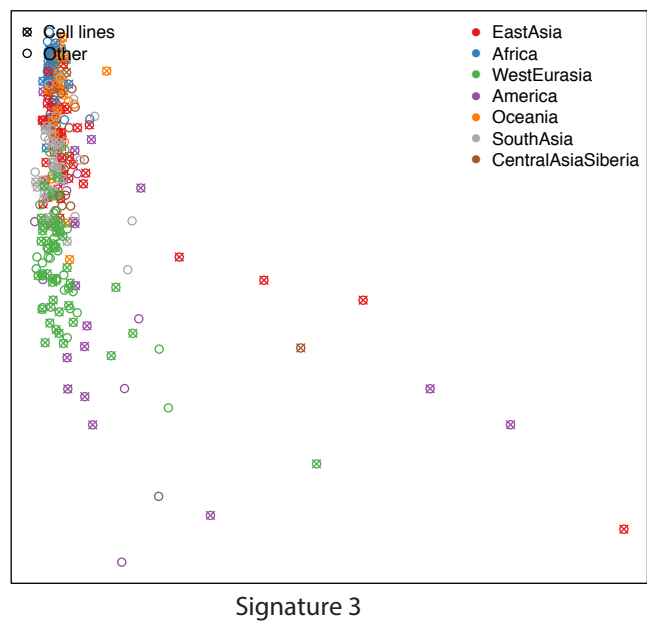**C**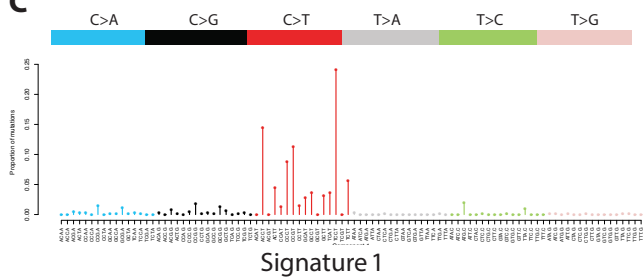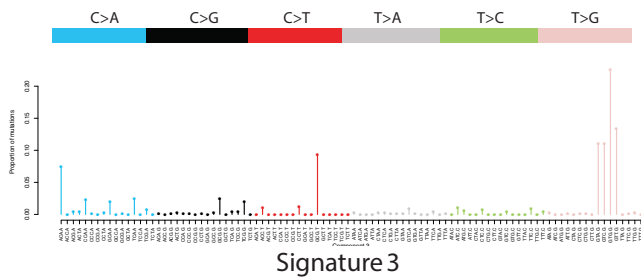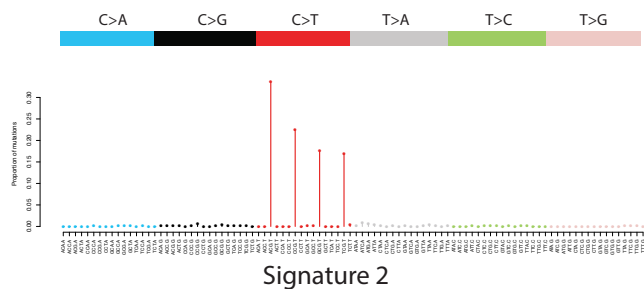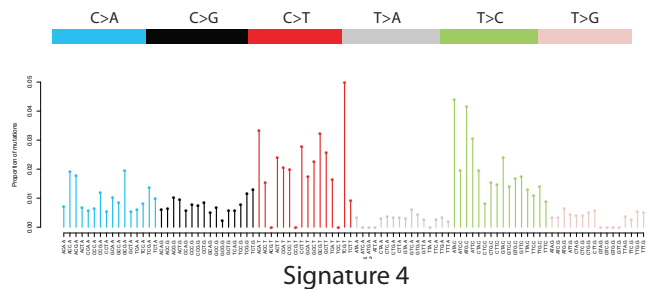

Supplement: S5 Fig — (PDF) [file pgen.1006581.s005.pdf]

A

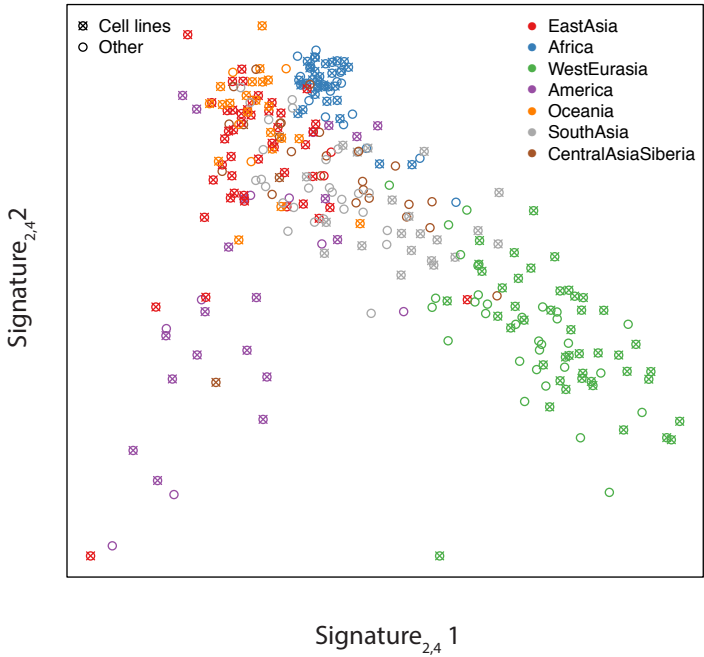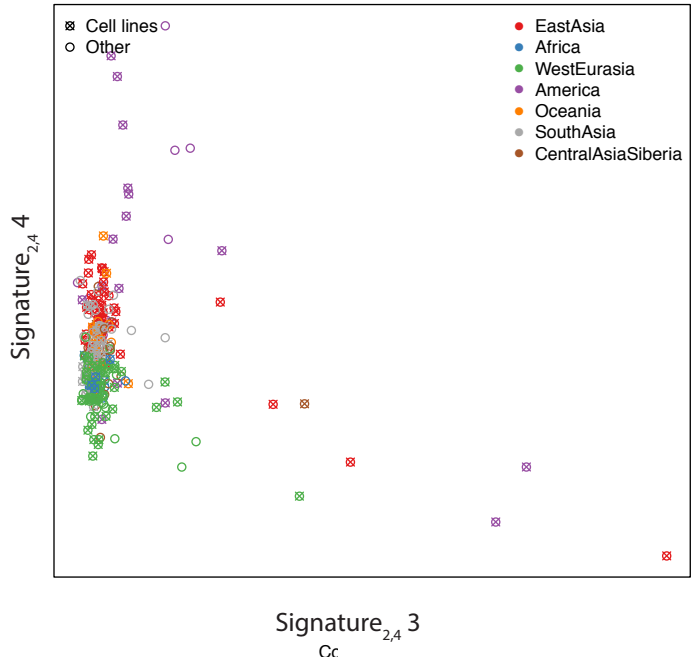

B

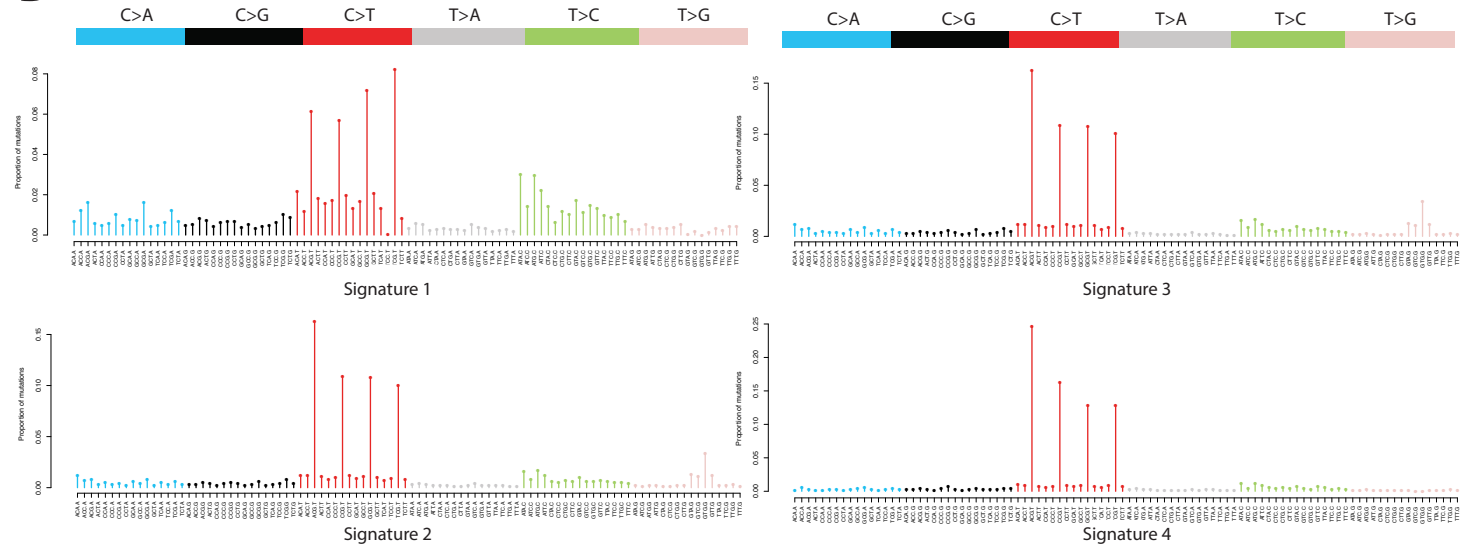

C

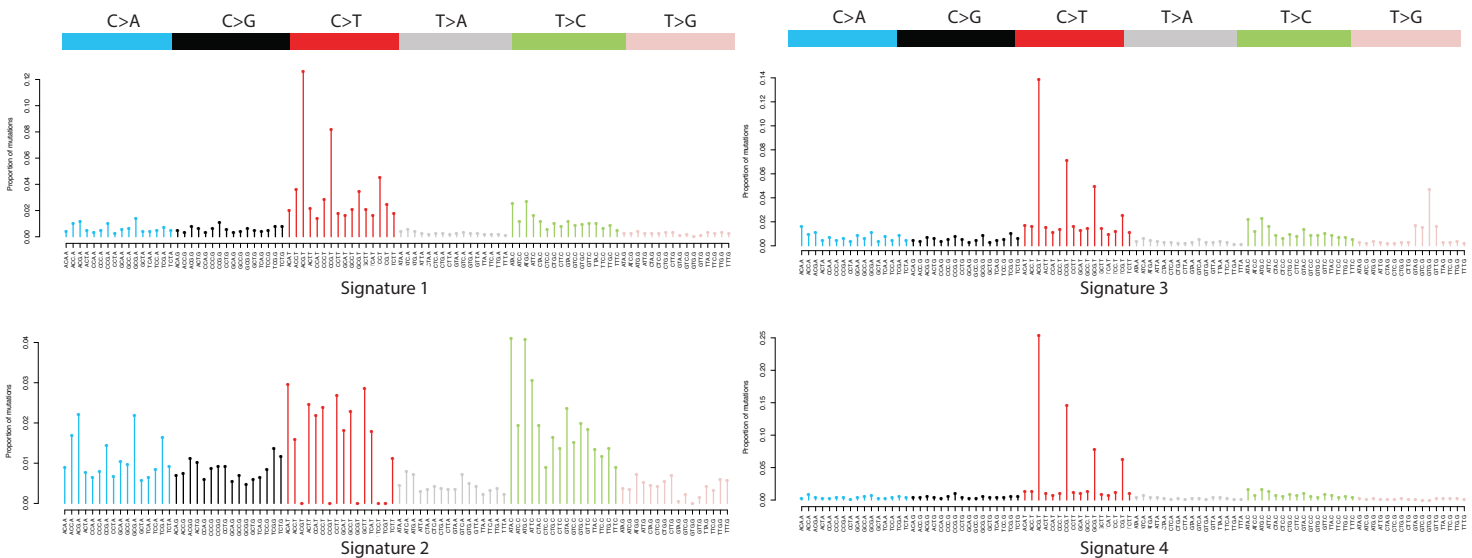

Supplement: S6 Fig — A: Distribution of signatures across samples. B: Mutational signatures 1–4. C: Mutational signatures 1–4 where, for each CpG mutation class, we subtracted the minimum over all four signatures from the signature. (PDF) [file pgen.1006581.s006.pdf]
